# The influence that different urban development models has on PM_2.5_ elemental and bioaccessible profiles

**DOI:** 10.1038/s41598-019-51340-4

**Published:** 2019-10-16

**Authors:** Gabriela Polezer, Andrea Oliveira, Sanja Potgieter-Vermaak, Ana F. L. Godoi, Rodrigo A. F. de Souza, Carlos I. Yamamoto, Rita V. Andreoli, Adan S. Medeiros, Cristine M. D. Machado, Erickson O. dos Santos, Paulo A. de André, Theotonio Pauliquevis, Paulo H. N. Saldiva, Scot T. Martin, Ricardo H. M. Godoi

**Affiliations:** 1 Environmental Engineering Department, Federal University of Parana, Curitiba, Parana, Brazil; 2 Chemistry Department, Federal University of Parana, Curitiba, Parana, Brazil; 3 Ecology & Environment Research Centre, Department of Natural Science, Manchester Metropolitan University, Manchester, M1 5GD United Kingdom; 4 Molecular Science Institute, University of the Witwatersrand, Johannesburg, South Africa; 5 Amazonas State University, Superior School of Technology, Manaus, Amazonas Brazil; 6 Chemical Engineering Department, Federal University of Parana, Curitiba, Parana, Brazil; 7 Postgraduate Program in Climate and Environment (CLIAMB, INPA/UEA), Manaus, Amazonas Brazil; 8 Department of Chemistry, Institute of Exact Sciences, Federal University of Amazonas, Manaus, Brazil; 9 Department of Pathology, LPAE (Air Pollution Lab), Faculty of Medicine, University of São Paulo, São Paulo, Brazil; 10 Department of Environmental Sciences, Federal University of Sao Paulo, Diadema, Brazil; 11 School of Engineering and Applied Sciences, Harvard University, Cambridge, MA United States

**Keywords:** Environmental sciences, Atmospheric chemistry, Environmental social sciences

## Abstract

Limited studies have reported on *in-vitro* analysis of PM_2.5_ but as far as the authors are aware, bioaccessibility of PM_2.5_ in artificial lysosomal fluid (ALF) has not been linked to urban development models before. The Brazilian cities Manaus (Amazon) and Curitiba (South region) have different geographical locations, climates, and urban development strategies. Manaus drives its industrialization using the free trade zone policy and Curitiba adopted a services centered economy driven by sustainability. Therefore, these two cities were used to illustrate the influence that these different models have on PM_2.5_ *in vitro* profile. We compared PM_2.5_ mass concentrations and the average total elemental and bioaccessible profiles for Cu, Cr, Mn, and Pb. The total average elemental concentrations followed Mn > Pb > Cu > Cr in Manaus and Pb > Mn > Cu > Cr in Curitiba. Mn had the lowest solubility while Cu showed the highest bioaccessibility (100%) and was significantly higher in Curitiba than Manaus. Cr and Pb had higher bioaccessibility in Manaus than Curitiba. Despite similar mass concentrations, the public health risk in Manaus was higher than in Curitiba indicating that the free trade zone had a profound effect on the emission levels and sources of airborne PM. These findings illustrate the importance of adopting sustainable air quality strategies in urban planning.

## Introduction

The socioeconomics, geographical location and urban development strategy of cities have a profound impact on the air quality. The consequences of continuous urban development and growth are, loss of green areas, increased traffic density, energy demand, waste generation, and industrialisation. The combined effect of these consequences results in poor air quality, which evidently has a negative impact on human health^1^. Besides, people at the lower end of the socioeconomic scale tend to reside and work in areas where high levels of air pollution prevail, and they generally have less access to medical evaluation and/or care^2^.

In this context, the air quality in two Brazilian cities, with vastly different geographical locations, were studied. On the one hand, the city of Manaus (capital of Amazonas State) is in the Northern part of the country, land-locked, and surrounded by the largest expanse of undisturbed (practically 1,500 km in all directions) tropical forest (the Amazon) in the world. On the other hand, Curitiba (capital of Parana State), located in the South region of Brazil, close to the Atlantic coast and surrounded by small cities and an established industry zone.

The two cities also differ significantly in their urban development strategies. They both adopted a new urban planning strategy in the 1960’s, Curitiba’s driven by Jaime Lerner, implementing innovative, inexpensive, integrated, sustainable urban planning whilst keeping people at the centre of his strategy. As one of the fastest growing cities in Brazil in the middle 20th century, the city administration succeeded in overcoming challenges of urban growth by innovative urban architecture, including the creation of several parks and green areas, redesigned road networks for public transport, and improved waste management^3,4^. These interventions and strategies ensure lower anthropogenic emission levels for a city of its size and population density. Manaus, in contrast, under military dictatorship, instilled an industrial free-trade zone (FTZ), resulting in unprecedented growth, mostly unplanned and driven by external investors. Increased demands for infrastructure in Manaus, evidently resulted in increased anthropogenic emissions, which is described as an aggregated pollution plume that is carried westward by prevailing winds by the GoAmazon project^5^. The GoAmazon 2014/15 project has put itself at task to study this air mass present over the city, which is currently still surrounded by the pristine atmosphere of the forest, as it provides a unique opportunity to study the effect of human intervention on air quality^6^. For these reasons, the two cities offer an interesting backdrop for investigating the impact that air quality has on human health.

It is to be expected that the lack of, or implementation of different, mitigating strategies to combat emissions will result in different pollution sources and chemical composition of airborne particulate matter (APM). It is well-known that human exposure to APM is detrimental to health^7^. During the last decade, scientists started to investigate not only the link between APM mass concentrations and human health, but also its elemental profile, and its bioaccessible fraction (the fraction labile in artificial body fluids)^8^.

*In vitro* studies can be used to determine the bioaccessibility of the APM, which is influenced by its chemical-physical properties. Due to the particle size of APM (PM_2.5_ and smaller), inhaled fractions penetrate deep into the lung, where it can induce oxidative stress or trans-locate across the blood barrier into the circulatory system, affecting and accumulating in other organs^9^. Apart from the particle size, the residence time of inhaled particles are critical. Typically, mucociliary clearance mechanisms or translocation, clear 85% of particles within 24 hours from the bronchial tree airway^9,10^. In the alveolar region, macrophages phagocytosis (more acidic conditions of the inflammation processes) prevails as clearance mechanism. The deposition of APM in the respiratory system may create localized pulmonary toxicological response, such as inflammation, and consequently stimulate engulfment by lung macrophages in the respiratory system. Then, fine particles stimulate alveolar macrophages engulfment, and metal(loid) dissolution may take place within this acidic environment^11^. For *in vitro* purposes, the composition of the lung fluid is essential and artificial lysosomal fluid (ALF) is used for phagocytosis studies^12,13^. The ALF dissolution profiles of PM_2.5_ can be seen as the maximum personal risk upon inhalation exposure, as its acidic nature enhances solubility by a factor of 2.5 to 8 times, for the elements tested, compared to the more neutral Gamble solution (see supplementary material). Lastly, the chemical composition and speciation of APM will determine dissolution rates and extent, which will clearly be influenced by pollution sources (economic activities), geographic and climate conditions, and mitigation strategies.

Although there are several *in vitro* studies in open literature focusing on the bioaccessible fraction of inhalable particles of different origin, exposed to various simulated lung fluid compositions^11^, only a handful investigated outdoor PM_2.5_ exposed to ALF. Wiseman & Zereini^14^ and Li *et al*.^15^ studied particles in Frankfurt, Germany and Nanjing, China, respectively. Although Luo *et al*.^16^ report on differences of the PM_2.5_ bioaccessibility in Gambles artificial lung fluid between 3 cities in China, the authors could not find any study linking bioaccessibility differences in ALF with urban development.

For that reason and reasons given previously, Manaus and Curitiba were chosen as two urban areas with similar populations, but different emission sources, environments and urban development strategies. We report on the differences in the bioaccessibility of Cu, Cr, Pb and Mn in outdoor PM_2.5_ after 1, 24 and 48 hours incubation periods in ALF between the two cities.

## Materials and Methods

### Study areas and PM_2.5_ sampling procedures

The geographical location and urban design of cities play an important part in their pollution budget. For that reason, two cities (Curitiba, Parana State and Manaus, Amazonia State) were chosen to illustrate the difference in risks to human health upon inhalation exposure to airborne particulates. Manaus, situated barely above sea level in the heart of the Amazon rainforest, has a tropical monsoon climate with an average temperature of 26.4 °C (ranging between 26.9 °C and 28.2 °C)^17^. Manaus is characterized by wet (summer) and dry (winter) seasons with annual average precipitation of 2145 mm and prevailing North-easterly winds all year round. Curitiba has an altitude of 924 meters above sea level, and although it is only 50 km from the Atlantic Ocean, a mountain range (1,800 meters) shields it from the marine atmosphere. It has a subtropical climate with a historic average temperature of 16.8 °C (ranging between 12.5 °C and 23.1 °C). The average annual precipitation is 1483 mm with no dry season and the prevailing wind direction is East and North-east^17^.

**Figure 1:**
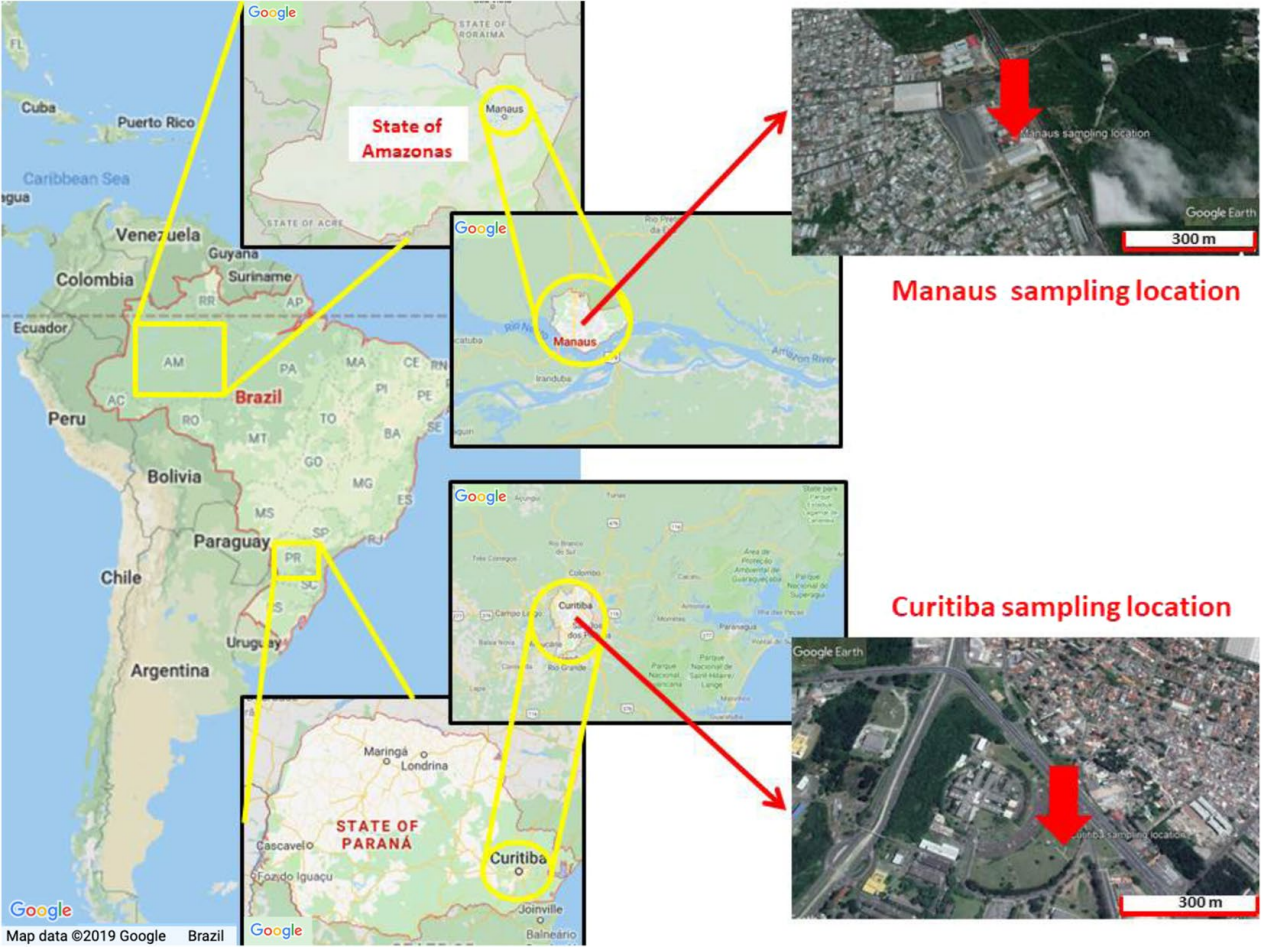
PM sampling locations in Manaus and Curitiba. The satellite map is from Google Maps (Map data©2019 Google; https://www.google.com/maps/place/Brasil); the satellite map is from Google Earth Pro (Map data©2019 Google; https://www.google.com/maps/@-10, -55.00001, 12646636 m/data = !3m1!1e3). The maps were edited with PowerPoint (version 16.28-19081202).

The PM_2.5_ samples were collected, at 2 m heights using low volume Harvard Impactor samplers operated at 10 L min^−1^ flow rates and 37 mm polycarbonate filters, in Manaus from August 2015 to August 2016 (15 samples; 72 h of sampling) and in Curitiba from November 2014 to July 2015 (24 samples; 168 h of sampling). The sampling took place during two consecutive years and could not be conducted simultaneously in both locations due to logistic constraints. However, with a large number of samples collected and the fact that no significant differences in seasonal variation of the concentration levels and bioaccessible profiles per city were observed, we are confident that the average levels reported are representative of the emission profiles of the two cities. Sampling sites were located in urban residential areas and the sampling locations illustrated in Fig. 1. In Manaus it was at the Boas Novas Faculty, in front of the Federal University of Amazonas (coordinates 3°6′12.5″ S 59°58′55.8″ O) and in Curitiba at the National Institute of Meteorology (INMET) station 25°26′93″ S, 49°13′85″ O), as illustrated in Fig. 1.

Apart from the clear difference in location, there are numerous other differences. These are: Area (the metropolitan area of Manaus is eleven times bigger than Curitiba, although with similar populations (2.1 and 1.9 million, respectively)^18^); Predominant traffic emissions (*Curitiba:* 1.5 million vehicles (2016) of which 69% cars, 9% motorcycles (using petrol and alcohol), 3% trucks (mainly diesel fuel); *Manaus:* 0.67 million vehicles (2016), of which 52% cars, 23% motorcycles, 11% trucks)^19^; Power supply (*Curitiba:* Hydropower >99%; Thermopower 14 MW; *Manaus:* Hydropower <15%; Thermopower 1,500 MW [28% coal and 72% gas^20,21^); Climate, etc. It is noteworthy to add that there are also differences in the gross domestic product (GDP) in that industry contributes 43% in the case of Manaus and only 23% in the case of Curitiba to GDP^22^. This would evidently lead to higher levels of anthropogenic emissions in Manaus, of which the chemistries would potentially be different from that in Curitiba.

### Bioaccessibility analysis

To determine the bioaccessible fraction of the element of interest, the PM_2.5_ samples were firstly analysed for its total elemental concentration by Energy Dispersive X-ray Fluorescence (EDXRF). Since this technique is non-destructive, the same sample could then be subjected to the *in vitro* study to determine the labile fraction in simulated lung fluid. The ratio leachate concentration and total elemental concentration provides the bioaccessible fraction. The elements of interest in this study were Cu, Cr, Pb and Mn due to their toxicity and carcinogenic properties.

### Total elemental concentration

The quantification of Cu, Cr, Pb and Mn in the PM_2.5_ samples were performed using a Minipal-4 (PANalytical) EDXRF, equipped with a Silicon Drift Detector (SDD) that is thermo-electrically cooled. Samples were analysed under He-atmosphere, with an acquisition time of 600 s, tube voltage of 30 kV, and a current of 0.3 mA. Calibration curves were obtained using reference standards (Micromatter, Seattle, WA, USA). The limit of detection (LOD) was calculated as three times the inverse of instrumental sensitivity multiplied by the square root of the background noise signal from the analysis of ten blank filters divided by the measurement time^23^.

The LOD’s obtained were 0.32 ng Cu m^−3^, 0.39 ng Cr m^−3^, 0.81 ng Pb m^−3^ and 0.35 ng Mn m^−3^. The method accuracy was 102% for Cu, 114% for Cr, 97% for Pb and 94% for Mn, and the precision measured as the relative standard deviation (RSD%) was 4% for Cu, 11% for Cr and Mn, and 3% for Pb obtained by the measurement of the NIST 2783 reference material (air particulate on filter media).

### *In vitro* procedure

Before analysis, all glass and plastic ware were immersed in 10% (v/v) HNO_3_ solution for 24 h, followed by rinsing with ultrapure water from Aquapur Evolution (resistivity of 18.2 MΩ cm). Six mL of freshly prepared ALF simulated lung fluid^13^ were added to each filter. These were placed in an incubator-shaker set to 37 °C and shaking at 40 cycles per minute. One mL samples were taken after 1, 24, and 48 hours, to be analysed with graphite furnace atomic absorption spectrometer (GFAAS).

### Cu, Cr, Mn and Pb soluble fraction determination

The leachates from the *in vitro* procedure were analysed with an AA 6800 GFAAS - Shimadzu, using background correction by deuterium lamp. Further information regarding the instrumental conditions and method development can be found in the Supplementary material Tables S1 and S2. The LOD and LOQ parameters were obtained respectively as three and ten times the standard deviation of ten replicate measurements of the simulated lung fluid ALF blank (no standards spiked) divided by the slope of the calibration curve for each element, the results were 0.4, 0.9, 2.9, 0.3 µg L^−1^ for the LOD; and for the LOQ were 1.5, 3.1, 9.8, 1.2 µg L^−1^ for Cu, Cr, Pb and Mn, respectively. The accuracy and repeatability were tested by repeatedly measuring spiked ALF solutions under the same conditions. Recoveries reported 82 and 109% for the 4 metals and the repeatability was within a suitable range, RSD < 10%^24^.

## Results and discussion

### Intercity differences of Manaus and Curitiba

**Figure 2:**
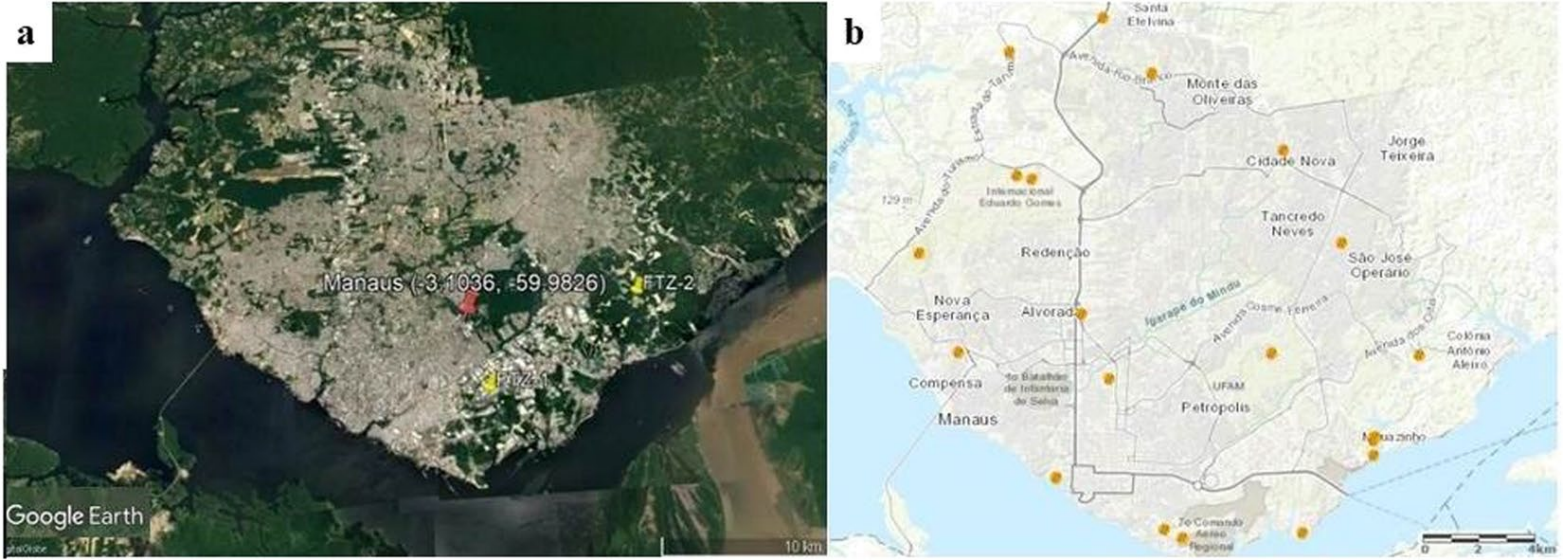
PM_2.5_ sampling location (red), industrial region (yellow) and the thermoelectric powerplants (orange) in Manaus. (**a**) The satellite map is from Google Earth Pro (Map data©2019 Google; https://www.google.com/maps/@-10, -55.00001, 12646636 m/data = !3m1!1e3); (**b**) Map obtained from the Electrical Sector Geographic Information System (SIGEL) of Brazil (https://sigel.aneel.gov.br/portal/home/), and available in the SIGEL website through the ESRI ArcMap 10.6.1 portal (https://sigel.aneel.gov.br/portal/portalhelp/en/website/help/#/What_s_new_in_Portal_for_ArcGIS_10_6_1/0193000000ws000000/).

**Figure 3:**
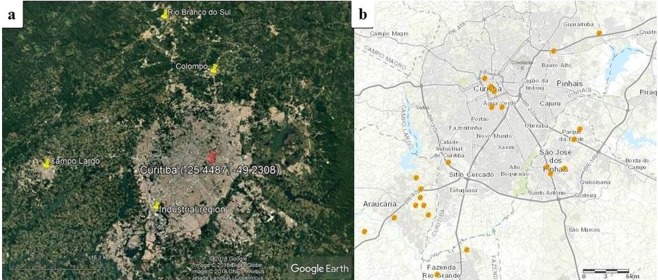
PM_2.5_ sampling location (red), industrial region (yellow) and the thermoelectric power plants (orange) in Curitiba. (**a**) The satellite map is from Google Earth Pro (Map data©2019 Google; https://www.google.com/maps/@-10,-55.00001,12646636 m/data = !3m1!1e3); (**b**) Map obtained from the Electrical Sector Geographic Information System (SIGEL) of Brazil (https://sigel.aneel.gov.br/portal/home/), and available in the SIGEL website through the ESRI ArcMap 10.6.1 portal (https://sigel.aneel.gov.br/portal/portalhelp/en/website/help/#/What_s_new_in_Portal_for_ArcGIS_10_6_1/0193000000ws000000/).

In this study, it was found that the PM_2.5_ average mass concentrations of samples for both cities were similar and relatively low (9.23 µg m^−3^ and 9.21 µg m^−3^, respectively for Curitiba and Manaus). Given that Manaus is located in a pristine area of the Amazon, one would have expected it to have a much lower mass concentration than Curitiba. Clearly, location seems not to explain the similar results. We have already alluded to the differences in some of the economic, urban development and expansion, and social features between Manaus and Curitiba. Figures 2 and 3 present the PM_2.5_ sampling location, industrial region, and the thermoelectric power plants locations in Manaus and Curitiba, respectively.

An essential part of these emissions will be transport related. River shipment is the main means of freight transport in Manaus and it is well-known that the harbour is the biggest floating dock in the world^25^. Marine emissions are then considered an important pollution source and contribute to the PM budget in Manaus, however, Cu, Cr, Mn and Pb have been found as not significant in emissions from ships^26,27^. Cargo transport in Curitiba, on the other hand, occurs mainly by road (specifically mainly by means of heavy vehicle transport)^28^ and to a lesser extent by rail and air. Transport emissions are known to contain the metals investigated in this study; Cu and Pb from fuel combustion exhaust^29^, Cu from the resuspension of asphalt and concrete from road surfaces^12^ Cr and Pb from road paint^14,30^, Pb emissions due to the legacy of leaded gasoline^14^, Cu from rubber tires and brake linings^12,14,31^, and Pb from vehicle wear and tear^14,31^.

A second, but equally important, a contributor to emissions in both cities is power generation. Diesel (28%) and natural gas (72%) fuel operated Thermal Power Plants (TPP) mainly generate the electricity supply in Manaus (Fig. 2b)^20,21^, with a 100 times higher wattage supply than in Curitiba. In Curitiba, on the other hand, almost all energy is supplied by hydroelectric plants (>99%). Therefore, it is expected that emissions from this source will be more in Manaus and have a different composition than in Curitiba.

**Figure 4:**
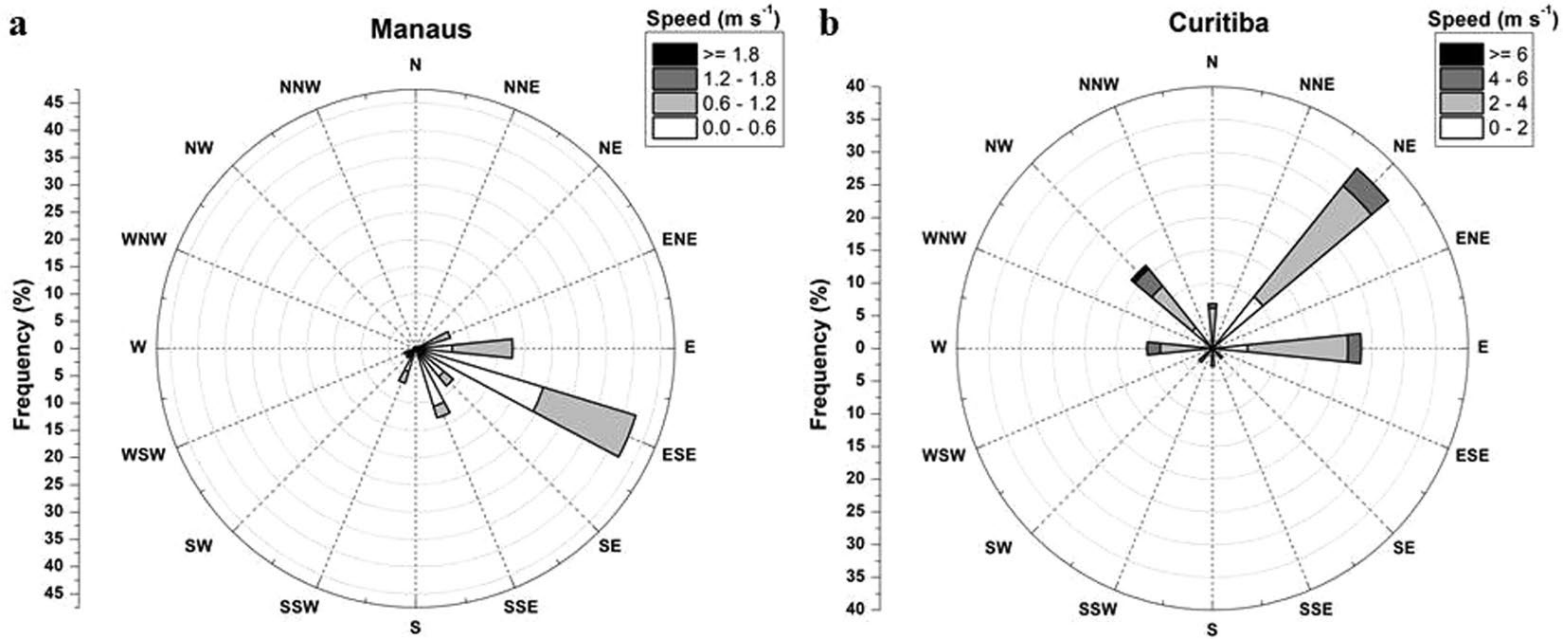
Wind roses of Manaus and Curitiba for the sampling periods.

The Free Trade Zone industry region is mostly located to the eastern and southeastern side of Manaus city centre (Fig. 2a), upwind to the city centre where sampling took place (Fig. 4). However, some industries can also be found in the northern area of the city. A large part of the almost 500 industries^32^ are found in this industrial zone and consist mainly of transport (motorcycles) and electronic technology manufacturers. These industries are all expected to contribute to the PM budget^15,33–38^.

The 27 industries located in the industrial zone of Curitiba are mainly automobile manufacturers and hi-tech industries^39,40^ and one petrochemical industry^41^. These are located in the southwest (Fig. 3a), downwind from the city centre and sampling point, as can be observed from the wind rose describing the predominant wind direction during the sampling period in Fig. 4. Although the contribution to the PM budget is expected to be low from this industrial region of Curitiba, there could be a substantial contribution from regional industries in the metropolitan area. The cities Colombo, Rio Branco do Sul and Campo Largo are located northwest and upwind of the city of Curitiba (Figs 3a and 4). Their industries contribute to the elemental composition of PM_2.5_ due to the combustion of coal in the limestone kilns^42,43^; fossil fuel, tires and industrial wastes in cement kilns, all providing sources for Mn, Cu, Pb and Cr^29,34,36,44,45^.

### Trace elemental concentrations

The total Cu, Mn, Cr and Pb concentrations in the PM_2.5_ samples were determined by EDXRF analysis, using the protocol described previously, and are presented in Table 1 as ng m^−3^. The standard deviation illustrates the significant variability of environmental samples, as observed in various studies before. The uncertainty is the propagation of uncertainty of measurements.

**Table 1:** Total and ALF leachate (1, 24 and 48 hours incubation period in ALF simulated lung fluid) atmospheric concentrations (ng m^−3^) obtained for Cu, Mn, Cr and Pb in PM_2.5_ samples collected in Manaus and Curitiba.

Atmospheric concentration (ng m^−3^)						
			Total	Soluble		
				1 h	24 h	48 h
**PM**_**2**. **5**_	**Curitiba**	average	**9**.**23**			
		S.D.	3.87			
	**Manaus**	average	**9**.**21**			
		S.D.	3.45			
**Copper**	**Curitiba**	average	**2**.**16**	*1*.*15*	*1*.*26*	*1*.*43*
		S.D.	0.90	0.35	0.42	0.46
		uncertainty	0.04	0.01	0.01	0.01
	**Manaus**	average	**10**.**56**	*2*.*34*	*2*.*44*	*2*.*76*
		S.D.	7.34	2.21	1.94	2.42
		uncertainty	0.22	0.02	0.02	0.01
**Manganese**	**Curitiba**	average	**4**.**28**	*0*.*64*	*0*.*70*	*0*.*70*
		S.D.	1.15	0.11	0.10	0.11
		uncertainty	0.02	0.00	0.00	0.01
	**Manaus**	average	**19**.**87**	*2*.*52*	*2*.*25*	*2*.*38*
		S.D.	14.30	1.89	1.83	1.79
		uncertainty	0.15	0.05	0.06	0.04
**Chromium**	**Curitiba**	average	**1**.**74**	*0*.*36*	*0*.*43*	*0*.*45*
		S.D.	1.22	0.21	0.31	0.32
		uncertainty	0.05	0.01	0.02	0.02
	**Manaus**	average	**3**.**16**	*1*.*32*	*1*.*30*	*1*.*53*
		S.D.	2.88	1.01	1.03	1.07
		uncertainty	0.22	0.04	0.05	0.07
**Lead**	**Curitiba**	average	**8**.**05**	*4*.*54*	*4*.*74*	*4*.*73*
		S.D.	8.57	4.31	4.78	4.92
		uncertainty	0.13	0.02	0.03	0.05
	**Manaus**	average	**12**.**15**	*8*.*47*	*7*.*90*	*8*.*31*
		S.D.	9.99	6.53	6.08	6.49
		uncertainty	0.55	0.13	0.23	0.14

The Cu, Cr and Mn concentrations in Curitiba where well below that of Manaus (~50% of the value in the case of Cu and Cr, and 25% of the value in the case of Mn) as expected (Manaus being industry driven due to the Free Trade Zone). The Mn levels in Manaus was the highest of all the potentially harmful elements (PHEs) tested (19.9 ng m^−3^) and Cr the lowest in Curitiba (1.74 ng m^−3^). The Pb levels are however similar between the two cities, and well below air quality guideline values^46^ of 500 ng m^−3^. The average total concentration levels in Curitiba followed the order Pb > Mn > Cu > Cr, but in Manaus it was Mn > Pb > Cu > Cr. Usmani and Kumar^45^ reported a similar order for the elemental concentrations (Mn > Cu > Pb > Cr) from fly ash emissions from 5 TPPs in India. They also found Mn levels much higher than the rest of the PTEs and ascribed that to the combustion emissions from the power generation plant. Research has shown that TPP emissions are a significant source of Mn, Cu, Cr^29,45^ and Pb^15,29,34,44,45^. Similar emission profiles and levels were found in three TPPs in Brazil^47^. Given that transport emissions from the marine origin are not significant contributors to these PTE levels, these findings suggest that the principal source for these four PTEs in Manaus are due to the TPP emissions, as predicted previously. On the other hand, for Curitiba, vehicle transport and regional industrial emissions (especially coal combustion) could be main contributors, as has been eluded to earlier.

Table 2 provides a summary of relevant literature, where the articles listed above the current data (in bold face) are generally reporting much higher concentration levels than found in Manaus or Curitiba, whilst the data below are representative of studies where similar concentrations are reported.

**Table 2:** Concentrations and sources of Cu, Cr, Pb and Mn in PM collected in different cities reported in open literature and compared to the present study.

City		Cu (ng m^−3^)		Cr (ng m^−3^)		Pb (ng m^−3^)		Mn (ng m^−3^)		PM_2.5_ (µg m^−3^)		ref.
		Conc.	Source	Conc.	Source	Conc.	Source	Conc.	Source	Conc.	source	
Algiers, Algeria	Major road	4600	Road dust/exhaust/ earth crust /rock rich in iron	55	Road dust/exhaust/earth crust/rock rich in iron	290	Oil combustion/road dust/exhaust (leaded gasoline)	5100	Road dust/ exhaust/earth crust/rock rich in iron	32		^48^
Algiers, Algeria	Urban site	750	Soil/road dust	25	Road dust/exhaust/earth crust/rock rich in iron	450	Oil combustion/road dust/exhaust (leaded gasoline)	1500	Road dust/ exhaust/earth crust/rock rich in iron	31		
Agra, India	Sub-urban site	190	Brake wear/industries	309	Iron industries/fuel burning	320	Soil dust/biomass and coal combustion	58	Soil dust/iron industries/fuel burning	132		^49^
Shanghai, China	Commercial	50	Fuel combustion/ brake wear	25	Steel smelting	50	Coal combustion/ metallurgic industries	50	Steel smelting/fuel addictive	62		^50^
	Residential	100	Fuel combustion/ brake wear	50	Steel smelting	150	Coal combustion/ metallurgic industries	50	Steel smelting/fuel addictive	58		
	Industrial	150	Fuel combustion/ brake wear	50	Steel smelting	300	Coal combustion/ metallurgic industries	100	Steel smelting/fuel addictive	78		
Nanjing, China	Residential	—	—	—	—	88	Metallurgic smelters/TPP	—	—	172		
										117		15
		—	—	—	—	49	TPP	—	—	61		
	Urban	104	—	21	—	158	—	71	—	—		^51^
Shanghai, China	Urban site	—	—	—	—	90	Traffic/oil combustion	55	Traffic/oil combustion	123		^16^
Nanjing, China		—	—	—	—	150	Traffic	70	Traffic	96		
Guangzhou, China		—	—	—	—	100	Traffic	30	Coal combustion /industry	71		
**Curitiba**, **Brazil**	**Residential**	**2**.**2**	**Traffic/coal combustion**	**1**.**7**	**Traffic/cement production**	**8**.**05**	**Traffic/coal combustion**	**4**.**3**	**Coal combustion**	**9**.**2**		**Present study**
**Manaus**, **Brazil**	**Residential**	**10**.**6**	**TPP /traffic/ industries**	**3**.**1**	**TPP**	**12**.**1**	**TPP /traffic/industries**	**19**.**9**	**TPP /industries**	**9**.**2**		
Monterey, Mexico	Central area	523	Vehicular	3.8	Vehicular	28	Vehicular	30	Vehicular	52	Vehicular	^52^
Frankfurt, Germany	Central area	53	—	9.7	—	13	—	19	—	—	Traffic	^14^
Córdoba, Argentina	Central area	7	—	7.9	—	8.6	—	4.4	Soil dust	48		^53^
Lecce, Italy	Sub-urban site	4.4	—	—	—	5.5	—	4.9	—	18	Regional background	^54^

Talbi *et al*.^48^ reports on PM concentrations in Algiers, a Mediterranean coastal city with 3.5 million inhabitants (1.5 times more than the two cities of interest). The much higher concentrations could be due to the specific site chosen, which is close to heavy traffic, a construction site, a waste incineration plant, and an iron smelter. Agarwal *et al*.^49^ investigated in Agra, India, the city of the Taj Mahal, with similar population than Manaus and Curitiba. Again, this site was close to an area with high traffic density and industries (metal casting, rubber processing, oil refinery, glass making).

Huang *et al*.^50^, Hu *et al*.^51^ and Luo *et al*.^16^ investigated PM_2.5_ levels in China (Shangai, Nanjing, and Guangzhou) and found levels that were, in general, lower than those reported in Algiers and Agra, but still significantly higher than the levels we found in Manaus and Curitiba. The presence of metallurgical industries may be partially the reason for the higher levels observed, especially in the case of lead. To that end, Li *et al*.^15^ showed that the lead levels in PM_2.5_ decreased by nearly 50% when the metallurgical smelters were shut down (the Chinese government did this in an attempt to improve the air quality in China during the 2014 Youth Olympic Games). The authors concluded that the lead emissions were due to coal combustion, which increased significantly when metallurgic smelters were in operation as well.

Comparable levels were found in Monterey (4 million residents), Mexico^52^, Córdoba (1.3 million inhabitants), Argentina^53^, Lecce (95,000 inhabitants), Italy^54^, and Frankfurt, Germany (730,000 inhabitants in the city and 2.3 million in the urban region^14^). The source of these emissions was mainly assigned to traffic, leading us to discern that the primary source of the emissions in Curitiba is traffic related. It is not unreasonable to add that industrial emissions will be playing a role as well, since these four cities all have similar industrial activities to that in Curitiba.

### Bioaccessibility

**Figure 5:**
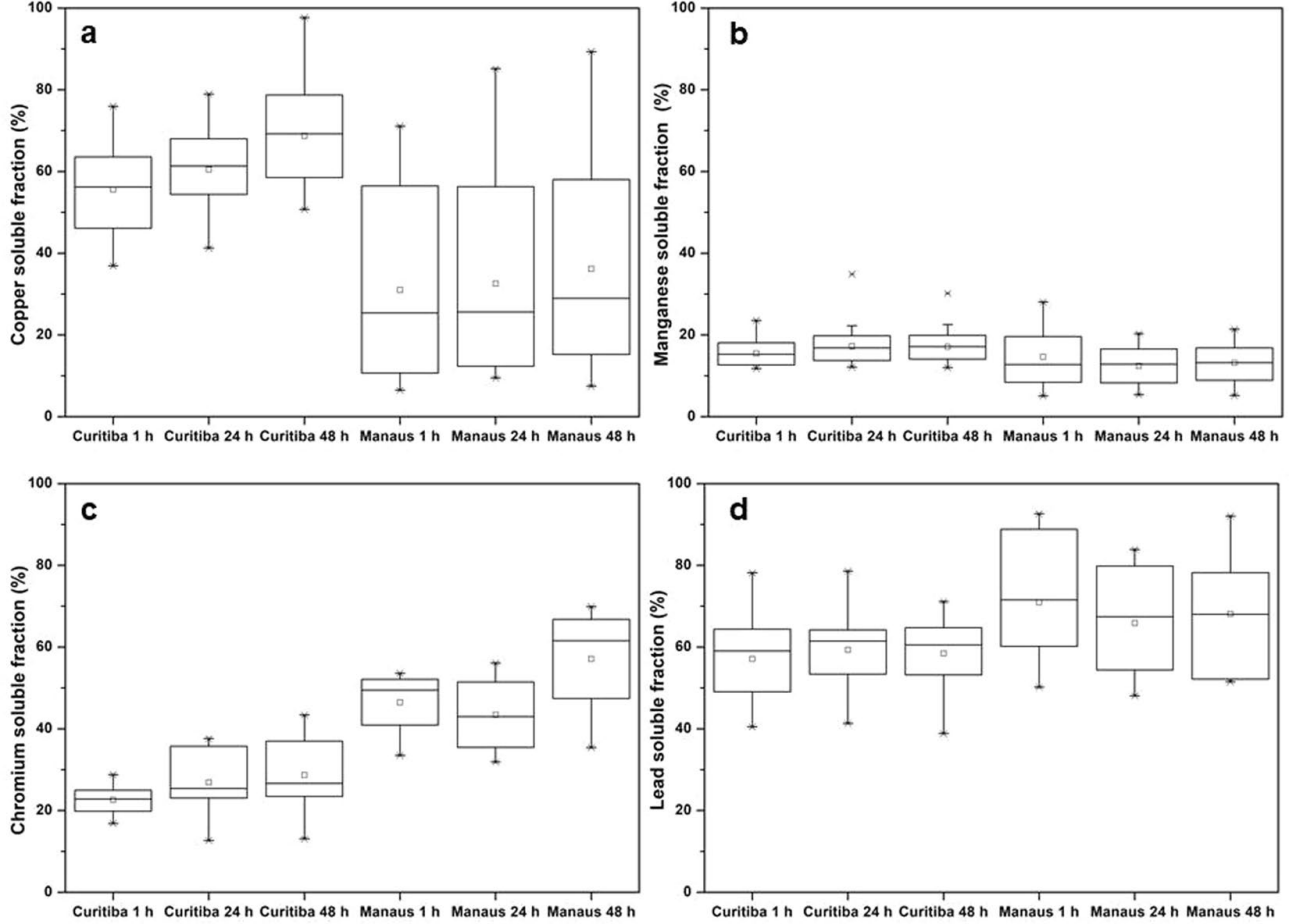
Boxplot graph of the accumulative bioaccessible fraction (%) of copper (**a**), manganese (**b**), chromium (**c**) and lead (**d**) extracted with ALF for 1, 24 and 48 hours of incubation period in Curitiba and Manaus PM_2.5_ samples.

The bioaccessible fraction in the ALF simulated lung fluid of Cu, Cr, Pb and Mn found in PM_2.5_ samples in Manaus and Curitiba are shown in Table 1. The absolute concentrations showed that the four PHEs were not very mobile in the ALF and displayed the following order: Pb > Mn > Cu > Cr in Manaus and Pb > Cu > Mn > Cr in Curitiba. The bioaccessibility was calculated as the cumulative fraction of the ALF-soluble concentration after 1, 24 and 48 hours of incubation and the total mass concentration. The averages represented as squares (ϒ) are presented in the boxplots of Fig. 5.

The percentage of bioaccessibility ranged from 10–65%, increased in general slightly over time, and differed between the two cities. It is noticeable that most of the leachable Pb and Cu were mobilised within the first hour. The high variability of the results for Cu, Cr and Pb can indicate the presence of different sources in both cities as these would most probably result in different chemistry and therefore also different solubilities. In general, the variability was higher in Manaus than in the Curitiba data for all elements. Cu showed the highest variability and varied; for example, from 7.5% to 89% (48 h) while for Pb, it varied from 51% to 92% (48 h). The lowest variability degree between the investigated compounds was obtained for Mn where it ranged from 12% to 30% (48 h) in Curitiba and in Manaus from 5.2% to 21% (48 h). This is indicative of a similar source (probably combustion).

The copper bioaccessibility was more than two times higher in Curitiba than in Manaus (Fig. 5a) and was the highest overall. In Manaus, it was also significantly lower than the 80% (24 h) found in Frankfurt^14^. We have already suggested that traffic emissions are one of the primary sources of Cu in Curitiba, similar to Frankfurt, whereas in Manaus the major source seems to be thermal power plants (Table 2). This is further corroborated by the findings of Wizeman & Zereini^14^ and Midander *et al*.^12^ where they also report enhanced Cu leaching when the sources are from vehicles exhaust emissions, brake lining attrition and rubber tires. The lower Cu bioaccessibility at Manaus could be due to the matrix in which the Cu is present, as the fuel used in the TPPs are heavily polluted oils or diesel. The diverse nature of industries in Manaus and the oil-fired TPPs will most probably cause emissions to be complex in composition. It can only be assumed that the Cu is locked in a matrix that is much less soluble than the Cu emissions in Curitiba which would probably be simple inorganic copper compounds or metallic Cu. The highly mobile Cu in the Curitiba samples are of a health concern. Charrier & Anastasio^55^ showed that Cu makes a significant contribution to the hydroxyl radical and HCOOH production upon PM inhalation and concludes that Cu is the most important contributor to the production of reactive oxygen species.

The lowest extracted bioaccessible fractions (14–18, and 11–17% for Curitiba and Manaus, respectively) between the investigated compounds were obtained for Mn (Fig. 5b). Fuel combustion from thermal power plants might be the major sources of Mn in Manaus whereas fossil fuel combustion of ceramics, limestone and cement kilns from the metropolitan cities surrounding Curitiba, contribute to Mn emissions in Curitiba^29,30,45^. The similar bioaccessibility profiles may be indicative of similar chemistry and chemical speciation of the Mn species found in the emissions. Comparing the bioaccessibility with that found in Frankfurt (52% (24 h)^14^), it is clear that the chemistry of the Mn between the two cities are different and therefore one would expect different sources. They have indicated that the primary source for Mn in Frankfurt was traffic-related, whilst we speculate that it is mainly combustion driven in our study.

Cr (Fig. 5c) showed an opposite behaviour to Cu with two times higher soluble fraction in Manaus than in Curitiba, ranging from 21–26% in Curitiba and 40–56% in Manaus. The emission sources and the consequent chemistry of the Cr will play a significant role. The chromium soluble fraction average was 23% (1 h), 27% (24 h), 29% (48 h) in Curitiba and 47% (1 h), 43% (24 h), 57% (48 h) in Manaus. Hexavalent chromium is generally considered as water-soluble^56^. Furthermore, Cr (VI) translocate easier from the lungs into the bloodstream than the Cr (III)^38^, which results in higher bioaccessibility in the lung fluid. Combustion processes would normally oxidize a fraction of Cr (III) to the hexavalent form. It is therefore interesting that the soluble Cr fraction in Curitiba was lower than what was expected, especially due to the close proximity of atmospheric Cr emitters: a cement plant and thermal power generation^37,38,57–59^. However, we should consider the trivalent Cr atmospheric budget in Curitiba^58^ for example from the road dust and 18,000 motor vehicles per day on the highway BR277 (40–45% of trucks)^60^. The higher ratio of Cr (III)/ Cr_total_ in Curitiba than Manaus may decrease the bioaccessibility of this element in Curitiba. Another factor to consider is that the increase of distance between the direct source of Cr (VI) and the sampling position would inevitably result in a reduction to Cr (III) compounds^37,38,56^. The distance between the cement industry plant and the sampling location in Curitiba is about 30 km, while in Manaus the distance between the TPPs and the sampling point range from 5 to 15 km. It is then possible, that a higher fraction of the hexavalent Cr emitted in the cement plant is reduced to Cr (III) in relation to the TPPs in Manaus, before reaching the sampling point. This hypothesis is corroborated by the results (31% in 24 h) obtained by Wiseman & Zereini^14^ for the bioaccessible fraction of Cr in Frankfurt, where traffic was the main Cr source (32,500 motor vehicles per day). It seems that Cr emitted from traffic sources generate Cr species that are lower in solubility and that Cr (VI) emissions are subjected to reduction during long-range transport. The results, therefore, indicate that the TPPs and other industries in Manaus are sources that generate the highest Cr soluble species impacting greatly on Cr bioaccessibility.

Pb showed the highest bioaccessible fraction on average between the investigated chemical elements with averages of 57% (1 h), 59% (24 h), 58% (48 h) in Curitiba, and 71% (1 h), 70% (24 h), 71% (48 h) in Manaus. It is also evident that the leaching is constant and independent of time. Wiseman & Zereini^14^ reported a much higher mobility for Pb from the Frankfurt PM samples (84%) and Li *et al*.^15^ reported 66% and 78% (24 h) for metallurgic smelters and coal combustion power plants in operation, respectively, and 61% (24 h) for only coal combustion power plants in operation in Nanjing. Although there are similarities between some Pb sources in Curitiba and Manaus (lime and cement kilns and TPPs) and Nanjing, and Frankfurt (traffic emissions and road dust), some differences in solubility are still observed. Given the site-specific chemistry of PM in general, these relatively small differences could be expected. The high lead bioaccessibility is a prominent health concern due to lead’s tendency to accumulate in bones^34^, where long term and continuous exposure can lead to severe toxicity in humans.

### Inhalable bioaccessible fraction in perspective

The hypothetical scenario discussed in the previous section eluded to different risks for two of the metals (Cr and Cu) between the two cities. To determine the overall risk, we calculated the daily inhaled bioaccessible fraction for two exposure groups: children between the ages of 1 and 11 years, and those older than 11 years (designated as adults). For that purpose, we used an average respiratory volume for the moderate activity level of 0.020 m^3^ min^−1^ for children and 0.026 m^3^ min^−1^ for adults^61^. The concentration we used was the one-hour bioaccessible fraction in each case since we found that between 80 and 100% of the element becomes mobile within the first hour of incubation. The average mass intake obtained and the main health risks associated with each chemical element investigated are presented in Table 3. As it was expected, the inhaled bioaccessible fraction was higher in Manaus than in Curitiba, since higher concentrations prevailed in Manaus. This can most likely be ascribed to the different urban development strategies followed, as discussed in the introduction.

**Table 3:** The average daily inhaled bioaccessible fraction (ng day^−1^) of Pb, Cr, Cu and Mn from urban fine PM_2.5_ in Curitiba and Manaus for children (1 to 11 years) and adults (>11 years).

Element	City	Average Inhaled bioaccessible fraction (ng day^−1^)		Main health effects	ref.
**Age group (years)1 to 11 > 11**					
**Pb**	**Curitiba** **Manaus**	129 242	173 323	Bone and Brain toxicity Damage in the nervous system, from intellectual development diminished in kids and deficit in performance in adults, to irreversible severe brain damage and death.	^34^
**Cr**	**Curitiba** **Manaus**	10 38	14 50	Carcinogenic Acute Cr (VI) exposure results in the respiratory tract injury, and for chronic exposure is considered human carcinogenic in inhalation route.	^38^
**Cu**	**Curitiba** **Manaus**	33 67	44 89	Oxidative stress Affects hepatic, gastrointestinal, nervous systems mainly for high concentrations; Potential to oxidize and induce the formation of reactive oxygen species (ROS)	^35^
**Mn**	**Curitiba** **Manaus**	18 71	24 94	Neurotoxicity Manganism: progressive, disabling neurological syndrome, with symptoms Parkinsonism-like. Also affects lungs and reproductive system.	^64^

The average mass of Cu and Pb inhaled in Manaus is about two times that of Curitiba. The deleterious effects of high respiratory exposure to Cu exacerbate the symptoms of Wilson’s disease, a rare hereditary disorder of the homeostasis regulation process of Cu, leading to high concentrations of Cu in body tissues^35^. In addition, Cu has been observed to have the potential to oxidize and induce the formation of reactive oxygen species (ROS), generating oxidative stress in the body^62,63^. As mentioned earlier, Charrier & Anastasio^55^ have indicated that inhaled Cu has the highest potential to cause permanent damage to DNA. Charrier & Anastasio^55^ determined the total hydroxyl radical production from soluble (in surrogate lung fluid) ambient concentrations as low as 0.88 ng/m^3^. Taking into account that our study reports values of 2.34 and 1.15 ng/m^3^ after one hour of incubation in Manaus and Curitiba, respectively, it is not unreasonable to deduce that long-term exposure could result in the **˙**OH production. As observed in other studies, we found that lead has a high soluble fraction in the simulated lung fluid^14,15^. Because of lead’s ability to accumulate in body tissue (especially in bones and teeth), its high toxicity at very low levels, and a half-life of 20 years, the findings of this study is of real concern. Although the values we reported in this study is not above the 500 ng/m^3^ level, it is expected that long-term low-dose exposure will have a profound effect on public health in both cities. More so in Manaus where a higher incidence of adverse health effects (sometimes irreversible damage in the nervous system and/or death) than in Curitiba^34^ is to be expected.

The average mass of Cr and Mn inhaled in Manaus is about four times that of Curitiba. Despite the fact that Mn is an essential body nutrient, long-term high respiratory exposure to Mn has been associated with a syndrome similar to Parkinson’s disease, where progressive permanent neurodegenerative damage is caused^64^. Studies conducted with animals (rodents and primates) have observed Mn accumulation in the brain due to slower elimination than from other organs^65^. Researchers have also observed a direct uptake through the nasal cavity to the brain through the olfactory nerve^65^. Additionally, some new studies suggested that the neurotoxicity of Mn may be associated with its interaction with iron (Fe) homeostasis, leading to high Fe deposition in the brain. The Fe accumulated in the brain generates cellular oxidative stress and neural damage^65^. Fe also has a synergistic effect on the hydroxyl radical production in conjunction with Cu^55^. Chromium, on the other hand, especially Cr (VI), can cause lesions on the respiratory tract and it is classified as a human carcinogen in the inhalation route^37^. The carcinogenic effect was suggested to happen during the chemical valence reduction from 6^+^ to 3^+^ by the generation of free radicals inside the cells, causing DNA damage^38^. Clearly, the public in Manaus is at greater toxic and carcinogenic risk than those in Curitiba.

It is evident from the results that elevated total elemental concentration values do not necessarily mean that the bioaccessibility will be high. It is therefore of importance to identify and quantify toxic and carcinogenic elements in APM and perform *in vitro* studies to determine the risk at the point of exposure.

This study has shown that the urban development strategies of two cities played a significant role in urban air quality. Although Manaus is in the so-called pristine Amazon, its free trade zone policy had a profound effect on the emission levels and sources of airborne PM, as well as the TPP. In contrast, Curitiba adopted innovative, inexpensive, integrated, sustainable urban planning with specific strategies to improve the well-being of its communities. Therefore, it is not surprising that both cities have shown PM_2.5_ mass concentrations that are nearly identical, despite the fact that Curitiba is not in a pristine environment. We have shown that the similar mass concentrations did not predict the differences in public health risk, that this study has revealed. We have shown that the public health risk between the two cities are different and that the risk in Manaus is overall is expected to be higher than in Curitiba. We propose that these differences observed are partially due to the different urban development strategies adopted by the two cities. We, therefore, point to the importance of considering public health and well-being when urban development strategies are formed.

## Supplementary information

The influence that different urban development models has on PM2.5 elemental and bioaccessible profiles
