# Supplementary material for: The influence that different urban development models has on PM2.5 elemental and bioaccessible profiles
Source: Sci Rep. 2019 Oct 16;9:14846. doi: 10.1038/s41598-019-51340-4 (PMC6795900; doi:10.1038/s41598-019-51340-4)
Supplement: Supplementary file 1 — The influence that different urban development models has on PM2.5 elemental and bioaccessible profiles [file 41598_2019_51340_MOESM1_ESM.docx]

**Supplementary Information SREP-19-14373A**

**The influence that different urban development models has on PM_2.5_ elemental and bioaccessible profiles**

**Gabriela Polezer^1^, Andrea Oliveira^2^, Sanja Potgieter-Vermaak^3,4^**^*^**, Ana F. L. Godoi^1^, Rodrigo A. F. de Souza^5^, Carlos I. Yamamoto^6^, Rita V. Andreoli^5^, Adan S. Medeiros^7^, Cristine M. D. Machado^8^, Erickson O. dos Santos^8^, Paulo A. de André^9^, Theotonio Pauliquevis^10^, Paulo H. N. Saldiva^9^, Scot T. Martin^11^, Ricardo H. M. Godoi^1^***

1. Environmental Engineering Department, Federal University of Parana, Curitiba, Parana, Brazil;
2. Chemistry Department, Federal University of Parana, Curitiba, Parana, Brazil;
3. Ecology & Environment Research Centre, Department of Natural Science, Manchester Metropolitan University, Manchester M1 5GD, United Kingdom;
4. Molecular Science Institute, University of the Witwatersrand, Johannesburg, South Africa;
5. Amazonas State University, Superior School of Technology, Manaus, Amazonas, Brazil;
6. Chemical Engineering Department, Federal University of Parana, Curitiba, Parana, Brazil;
7. Postgraduate Program in Climate and Environment (CLIAMB, INPA/UEA), Manaus, Amazonas, Brazil;
8. Department of Chemistry, Institute of Exact Sciences, Federal University of Amazonas, Brazil;
9. Department of Pathology, LPAE (Air Pollution Lab), Faculty of Medicine, University of São Paulo, São Paulo, Brazil;
10. Department of Environmental Sciences, Federal University of Sao Paulo, Diadema, Brazil
11. School of Engineering and Applied Sciences, Harvard University, 02138 Cambridge, MA, United States.

Correspondence to: Ricardo H. M. Godoi (rhmgodoi@ufpr.br) and Sanja Potgieter-Vermaak (S.Potgieter@mmu.ac.uk)

All measurements of Cu, Cr, Pb and Mn in the soluble fraction of the simulated lung fluid were carried out using an atomic absorption spectrometer (model AA 6800, Shimadzu), equipped with a background correction by deuterium lamp, and longitudinal heating with a pyrolytic coated graphite tube. Argon pure (99.9%) was employed as purge and protective gas. The spectrometer was operated with hollow cathode lamps (HCl). The measurement mode was obtained with the integrated absorbance, and the calibration was obtained with acidic standard concentration (HNO_3_ 1% (v/v)) in µg L^-1^. The samples and chemical modifier volumes employed were 20 μL and 5 μL respectively.

**Supplementary Table 1.** Instrumental parameters of GFAAS for the quantification of Cu, Cr, Mn and Pb in ALF.

| **Parameters** | **Cu** | **Cr** | **Mn** | **Pb** |
| --- | --- | --- | --- | --- |
| Measurement wavelength (nm) | 324.8 nm | 360.5 | 279.5 | 283.3 |
| Spectral bandpass (nm) | 0.5 | 0.5 | 0.2 | 1 |

Supplementary Table 2. Graphite furnace heating program for Cu, Cr, Mn and Pb for direct determination in simulated lung fluid ALF sample. a: 1100 °C (Cu), 1200 °C (Mn), 1550 °C (Cr), 1150 °C (Pb). b: 2100 °C (Cu), 2200 °C (Mn), 2400 °C (Cr), 2000 °C (Pb).

| **Step** | **Temperature (° C)** | **Ramp Time (s)** | **Hold Time (s)** | **Gas flow rate (L min^-1^)** |
| --- | --- | --- | --- | --- |
| Drying 1 | 85 | 5 | 0 | 0.1 |
| Drying 2 | 110 | 50 | 0 | 0.1 |
| Pyrolysis 1 | T_pyr_^a^ | 10 | 10 | 1 |
| Pyrolysis 2 | T_pyr_^a^ | 0 | 3 | 0 |
| Atomization | T_atom_^b^ | 0 | 3 | 0 |
| Clean | 2500 | 0 | 2 | 1 |

**Supplementary Table 3.** Bioaccessibility of Pb, Cu, Cr and Mn in simulated lung fluids.

| Simulated lung fluid | Incubation time | Size fraction (µm) | Matrix/origin | City, Country | Bioaccessible average % | | | Ratio ALF/Gamble | Reference |
| --- | --- | --- | --- | --- | --- | --- | --- | --- | --- |
|  |  |  |  |  | Others | ALF | Gamble |  |  |
| **Pb** | | | | | | | | | |
| ALF | 24 h | 0.5 | PM/industrial | Bolivia |  | 35/75 |  |  | 1 |
| Ammonium Acetate | 2 h | <1 | PM/urban | Ottawa, Canada | 15 |  |  |  | 2 |
| ALF/Gamble | 24 h |  | PM/urban | Brno, Czech Republic |  | 84 | 1 | 84.0 | 3 |
| ALF/Gamble | 24 h |  | PM/urban | Frankfurt, Germany |  | 78 | 5 | 15.6 | 4 |
| ALF/Gamble | 24 h | <2.5 | PM/urban | Frankfurt, Germany |  | 84 | 4 | 21.0 | 4 |
| ALF/Gamble | 24 h |  | PM/industrial | Nanjing, China |  | 66 | 10 | 6.6 | 5 |
| ALF/Gamble | 24 h |  | PM/industrial | Nanjing, China |  | 78 | 11 | 7.1 | 5 |
| ALF/Gamble | 24 h |  | PM/industrial | Nanjing, China |  | 61 | 20 | 3.1 | 5 |
| ALF/Gamble | 24 h |  | PM/urban | Singapore, Republic of Singapore |  | 45 | 7 | 6.4 | 6 |
| ALF/Gamble | 24 h |  | PM/urban | Singapore, Republic of Singapore |  | 43 | 8 | 5.4 | 6 |
| **PM_2.5_ ALF/GAMBLE average** | | | | |  | 62.8 | 10.0 | 8.3 |  |
| Gamble | 24 h | <2.5 | PM/urban-industrial | Guangzhou, China |  |  | 1 |  | 7 |
| Gamble | 24 h |  | PM/urban-industrial | Shanghai, China |  |  | 2 |  | 7 |
| Gamble | 24 h |  | PM/urban-industrial | Nanjing, China |  |  | 2 |  | 7 |
| Gamble | 24 h |  | PM/industrial | Dunkirk, France |  |  | 18 |  | 8 |
| Gamble | 24 h |  | PM/industrial | Dunkirk, France |  |  | 58 |  | 8 |
| Gamble | 24 h |  | PM/urban | Dunkirk, France |  |  | 39 |  | 8 |
| **PM_2.5_ average** | | | | |  | 62.8 | 14.6 | 8.3 |  |
| ALF/Gamble | 24 h | <3.3 | PM/indoor winter | Nanjing, China |  | 50 | 0.9 | 55.6 | 9 |
| ALF/Gamble | 24 h |  | PM/indoor spring | Nanjing, China |  | 60 | 4.3 | 14.0 | 9 |
| ALF/Gamble | 24 h |  | PM/outdoor winter | Nanjing, China |  | 42.5 | 0.8 | 53.1 | 9 |
| ALF/Gamble | 24 h |  | PM/outdoor spring | Nanjing, China |  | 58 | 2 | 29.0 | 9 |
| Gamble modified | 6 days | < 5 | Coal fly ash | Australia |  |  | 9 |  | 10 |
| Gamble/water | 24 h |  | NIST 1648a (PM) | United States | 4.4 |  | 14.5 |  | 11 |
| Gamble/water | 24 h | <10 | BCR38 (coal fly ash) | United Kingdom | 1.3 |  | 3.3 |  | 11 |
| Gamble modified | 24 h |  | PM/urban | Rio de Janeiro, Brazil |  |  | 11 |  | 12 |
| ALF/Gamble | 24 h |  | PM/traffic | Frankfurt, Germany |  | 96 | 26 | 3.7 | 4 |
| ALF | 1 h |  | PM | Graz, Austria and Karachi, Pakistan |  | 61 |  |  | 13 |

**Supplementary Table 3.** continued

| Simulated lung fluid | Incubation time | Size fraction (µm) | Matrix/origin | | City, Country | Bioaccessible average % | | | | | Ratio ALF/Gamble | Reference |
| --- | --- | --- | --- | --- | --- | --- | --- | --- | --- | --- | --- | --- |
|  |  |  |  | |  | Others | | ALF | Gamble | |  |  |
| **Pb** | | | | | | | | | | | | |
| ALF/Gamble | 24 h | 63-125 | Road dust | | Manchester, United Kingdom |  | | 46.5 | 0.3 | | 155.0 | 14 |
| Gamble/water | 24 h | TSP | Vehicle exhaust | |  | 18.7 | |  | 45.2 | |  | 11 |
| ALF/Gamble | 48 h |  | PM/urban-industrial | | Shanghai, China |  | | 58 | 8 | | 7.3 | 15 |
| Gamble/water | 24 h |  | NIST 2584 (vacuums cleaner dust) | | United States | 5.2 | |  | 24.6 | |  | 11 |
| Gamble/water | 24 h |  | NIST 2584 (vacuums cleaner dust) | | United States | 5.2 | |  | 24.6 | |  | 11 |
| Average | | | | | | 8.3 | | 63.1 | 12.8 | | 31.1 |  |
| **Cu** | | | | | | | | | | | | |
| ALF | 24 h | 0.5 | PM mine/smelting city | | Bolivia |  | | 70/60 |  | |  | 1 |
| Ammonium Acetate | 2 h | <1 | PM/urban | | Ottawa, Canada | 45-50 | |  |  | |  | 2 |
| ALF/Gamble | 24 h |  | PM/urban | | Brno, Czech Republic |  | | 75 | 22 | | 3.4 | 3 |
| ALF/Gamble | 24 h |  | PM/traffic | | Frankfurt, Germany |  | | 87 | 48 | | 1.8 | 4 |
| ALF/Gamble | 24 h | <2.5 | PM/traffic | | Frankfurt, Germany |  | | 80 | 31 | | 2.6 | 4 |
| ALF/Gamble | 24 h |  | PM/urban | | Singapore, Republic of Singapore |  | | 79 | 39 | | 2.0 | 6 |
| ALF/Gamble | 24 h |  | PM/urban | | Singapore, Republic of Singapore |  | | 83 | 30 | | 2.8 | 6 |
| **PM_2.5_ ALF/GAMBLE average** | | | | | |  | | 80.7 | 33.3 | | 2.5 |  |
| Gamble | 24 h |  | PM/industrial | | Dunkirk, France |  | |  | 68 | |  | 8 |
| Gamble | 24 h |  | PM/smelters | | Dunkirk, France |  | |  | 76 | |  | 8 |
| Gamble | 24 h |  | PM/urban | | Dunkirk, France |  | |  | 79 | |  | 8 |
| **PM_2.5_ average** | | | | | |  | | 80.7 | 50.9 | | 2.5 |  |
| ALF/Gamble | 24 h | <3.3 | PM/indoor winter | Nanjing, China | |  | 27.5 | | 24.3 | 1.1 | | 9 |
| ALF/Gamble | 24 h |  | PM/indoor spring | Nanjing, China | |  | 26.3 | | 27.2 | 1.0 | | 9 |
| ALF/Gamble | 24 h |  | PM/outdoor winter | Nanjing, China | |  | 23.5 | | 24.3 | 1.0 | | 9 |
| ALF/Gamble | 24 h |  | PM/outdoor spring | Nanjing, China | |  | 25.2 | | 24 | 1.1 | | 9 |
| Gamble modified | 6 days | < 5 | coal fly ash | Australia | |  |  | | 33 |  | | 10 |
| Gamble/water | 24 h |  | NIST 1648a (PM) | United States | | 20.4 |  | | 41.3 |  | | 11 |
| Gamble/water | 24 h | <10 | BCR38(coal fly ash) | United Kingdom | | 4.3 |  | | 36.5 |  | | 11 |
| ALF | 1 h |  | PM | Graz, Austria and Karachi, Pakistan | |  | 26 | |  |  | | 13 |
| ALF/Gamble | 24 h |  | PM/traffic | Frankfurt, Germany | |  | 83 | | 40 | 2.1 | | 4 |
| Gamble modified | 24 h |  | PM/urban | Rio de Janeiro, Brazil | |  |  | | 18 |  | | 12 |

**Supplementary Table 3.** continued

| Simulated lung fluid | Incubation time | Size fraction (µm) | Matrix/origin | City, Country | Bioaccessible average % | | | Ratio ALF/Gamble | Reference |
| --- | --- | --- | --- | --- | --- | --- | --- | --- | --- |
|  |  |  |  |  | Others | ALF | Gamble |  |  |
| **Cu** | | | | | | | | | |
| ALF/Gamble | 48 h | TSP | PM/urban-industrial | Shanghai, China |  | 65 | 37 | 1.8 | 15 |
| Gamble/water | 24 h |  | Vehicle exhaust |  | 8.5 |  | 38.5 |  | 11 |
| Gamble/water | 24 h |  | NIST 2584 (vacuum cleaner dust) | United States | 17.6 |  | 40.9 |  | 11 |
| Average | | | | | 12.7 | 60.1 | 39.2 | 1.9 |  |
| **Cr** | | | | | | | | | |
| ALF/Gamble | 24 h | <1 | PM/traffic | Frankfurt, Germany |  | 61 | 21 | 2.9 | 4 |
| ALF/Gamble | 24 h |  | PM/urban | Brno, Czech Republic |  | 24 | 20 | 1.2 | 3 |
| ALF/Gamble | 24 h | <2.5 | PM/traffic | Frankfurt, Germany |  | 31 | 9 | 3.4 | 4 |
| ALF/Gamble | 24 h |  | PM/urban | Singapore, Republic of Singapore |  | 44 | 14 | 3.1 | 6 |
| ALF/Gamble | 24 h |  | PM/urban | Singapore, Republic of Singapore |  | 47 | 19 | 2.5 | 6 |
| **PM_2.5_ ALF/GAMBLE average** | | | | |  | 40.7 | 14.0 | 3.0 |  |
| Gamble modified | 6 days | < 5 | coal fly ash | Australia |  |  | 0.08 |  | 10 |
| ALF/Gamble | 24 h | <10 | PM/traffic | Frankfurt, Germany |  | 31 | 21 | 1.5 | 4 |
| Gamble modified | 24 h |  | PM/urban | Rio de Janeiro, Brazil |  |  | 2 |  | 12 |
| ALF/Gamble | 4 weeks | 63-125 | Road dust | Manchester, United Kingdom |  | 19.3 | 1.6 | 12.1 | 14 |
| ALF/Gamble | 48 h | TSP | PM/urban-industrial | Shanghai, China |  | 44 | 19 | 2.3 | 15 |
| Average | | | | |  | 38.0 | 12.8 | 3.6 |  |
| **Mn** | | | | | | | | | |
| ammonium acetate | 2 h | <1 | PM/urban | Ottawa, Canada | 50-60 |  |  |  | 2 |
| ALF/Gamble | 24 h |  | PM/urban | Brno, Czech Republic |  | 63 | 0 |  | 3 |
| ALF/Gamble | 24 h |  | PM/traffic | Frankfurt, Germany |  | 63 | 8 | 7.9 | 4 |
| ALF/Gamble | 24 h | <2.5 | PM/urban | Singapore, Republic of Singapore |  | 39 | 27 | 1.4 | 6 |
| ALF/Gamble | 24 h |  | PM/urban | Singapore, Republic of Singapore |  | 30 | 33 | 0.9 | 6 |
| ALF/Gamble | 24 h |  | PM/urban | Frankfurt, Germany |  | 52 | 9 | 5.8 | 4 |
| **PM_2.5_ ALF/GAMBLE average** | | | | |  | 40.3 | 23.0 | 2.7 |  |

**Supplementary Table 3.** continued

| Simulated lung fluid | Incubation time | Size fraction (µm) | Matrix/origin | City, Country | Bioaccessible average % | | | Ratio ALF/Gamble | Reference |
| --- | --- | --- | --- | --- | --- | --- | --- | --- | --- |
|  |  |  |  |  | Others | ALF | Gamble |  |  |
| **Mn** | | | | | | | | | |
| Gamble | 24 h | <2.5 | Urban-industrial | Shanghai, China |  |  | 18 |  | 7 |
| Gamble | 24 h |  | PM/urban-industrial | Nanjing, China |  |  | 15 |  | 7 |
| Gamble | 24 h |  | PM/urban-industrial | Guangzhou, China |  |  | 10 |  | 7 |
| Gamble | 24 h |  | PM/industrial | Dunkirk, France |  |  | 16 |  | 8 |
| Gamble | 24 h |  | PM/industrial | Dunkirk, France |  |  | 40 |  | 8 |
| Gamble | 24 h |  | PM/urban | Dunkirk, France |  |  | 27 |  | 8 |
| **PM_2.5_ average** | | | | |  | **40.3** | **21.8** | **2.7** |  |
| ALF/Gamble | 24 h | <3.3 | PM/indoor winter | Nanjing, China |  | 45 | 5.7 | 7.9 | 9 |
| ALF/Gamble | 24 h |  | PM/indoor spring | Nanjing, China |  | 34.2 | 19 | 1.8 | 9 |
| ALF/Gamble | 24 h |  | PM/outdoor winter | Nanjing, China |  | 44.8 | 22.3 | 2.0 | 9 |
| ALF/Gamble | 24 h |  | PM/outdoor spring | Nanjing, China |  | 35.9 | 11.4 | 3.1 | 9 |
| Gamble/water | 24 h | 5 | NIST 1648a (PM) | United States | 24.5 | 52.2 |  |  | 11 |
| ALF/Gamble | 24 h | <10 | PM/urban | Frankfurt, Germany |  | 57 | 27 | 2.1 | 4 |
| ALF | 1 h |  | PM | Graz, Austria and Karachi, Pakistan |  | 65 |  |  | 13 |
| Gamble modified | 24 h |  | PM/urban | Rio de Janeiro, Brazil |  |  | 33 |  | 12 |
| Gamble/water | 24 h |  | BCR38 (coal fly ash) | United Kingdom | 6.4 | 32.3 |  |  | 11 |
| ALF/Gamble | 48 h | TSP | PM/urban-industrial | Shanghai, China |  | 32 | 20 | 1.6 | 15 |
| Gamble/water | 24 h |  | NIST 2584 (vacuum cleaner dust) | United States | 13.1 | 24.3 |  |  | 11 |
| **Average** | | | | | 14.7 | 44.1 | 19.3 | 3.5 |  |

**References**

1. Goix, S. et al. Metal concentration and bioaccessibility in different particle sizes of dust and aerosols to refine metal exposure assessment. J Hazard Mater. **317**, 552-562 (2016).
2. Niu, J., Rasmussen, P. E., Hassan, N. M., Vincent, R. Concentration Distribution and Bioaccessibility of Trace Elements in Nano and Fine Urban Airborne Particulate Matter: Influence of Particle Size. Water Air Soil Poll. **213**, 211-225 (2010).
3. Coufalík, P., Mikuska, P., Matousek, T., Vecera, Z. Determination of the bioaccessible fraction of metals in urban aerosol using simulated lung fluids. Atmos Environ. **140**, 469-475 (2016).
4. Wiseman, C. L. S. & Zereini, F. Characterizing metal(loid) solubility in airborne PM10, PM_2.5_ and PM1 in Frankfurt, Germany using simulated lung fluids. Atmos Environ. **89**, 282-289 (2014).
5. Li, S.-W. et al. Influence of pollution control on lead inhalation bioaccessibility in PM_2.5_: A case study of 2014 Youth Olympic Games in Nanjing. Environ Int. **94**, 69-75 (2016).
6. Huang, X., Betha, R., Tan, L-Y., Balasubramanian, R. Risk assessment of bioaccessible trace elements in smoke haze aerosols versus urban aerosols using simulated lung fluids. Atmos Environ. **125** 505–511 (2016).
7. Luo, X. et al. Pulmonary bioaccessibility of trace metals in PM_2.5_ from different megacities simulated by lung fluid extraction and DGT method. Chemosphere. **218,** 915-921 (2019).
8. Mbengue, S., Alleman, L. Y., Flament, P. Bioaccessibility of trace elements in fine and ultrafine atmospheric particles in an industrial environment. Environ Geochem Hlth. **37**, 875-889 (2015).
9. Tang, Z-J., Hu, X., Qiao, J.-Q., Lian, H.-Z. Size Distribution, Bioaccessibility and Health Risks of Indoor/Outdoor Airborne Toxic Elements Collected from School Office Room. Atmosphere. **9,** 340 (2018).
10. Twining, J., McGlinn, P., Loi, E., Smith, K., Gieré, R. Risk Ranking of Bioaccessible Metals from Fly Ash Dissolved in Simulated Lung and Gut Fluids. Environ Sci Technol. **39**, 7749-7756 (2005).
11. Julien, C., Esperanza, P., Bruno, M., Alleman, L. Y. Development of an in vitro method to estimate lung bioaccessibility of metals from atmospheric particles. J Environ Monitor. **13**, 621-630 (2011).
12. Da Silva, L.I.D. et al. Evaluation of bioaccessible heavy metal fractions in PM10 from the m0etropolitan region of Rio de Janeiro city, Brazil, using a simulated lung fluid. Microchem. J. **118**, 266–271 (2015).
13. Mukhtar, A.; Mohr, V.; Limbeck, A. The suitability of extraction solutions to assess bioaccessible trace metal fractions in airborne particulate matter: a comparison of common leaching agents. Environ Sci Pollut R. **22**, 16620-16630 (2015).
14. Potgieter-Vermaak, S., Rotondo, G., Novakovic, V., Rollins, S., Grieken, R.V. Component- specific toxic concerns of the inhalable fraction of urban road dust. Environ. Geochem. Health. **34**, 689–696 (2012).
15. Huang, X., Cheng, J., Bo D., Betha R., Balasubramanian R. Bioaccessibility of Airborne Particulate-Bound Trace Elements in Shanghai and Health Risk Assessment. Frontiers in Environmental Science. **4**, (2016).
